# IBI362 (LY3305677), a weekly-dose GLP-1 and glucagon receptor dual agonist, in Chinese adults with overweight or obesity: A randomised, placebo-controlled, multiple ascending dose phase 1b study

**DOI:** 10.1016/j.eclinm.2021.101088

**Published:** 2021-08-13

**Authors:** Linong Ji, Hongwei Jiang, Pei An, Huan Deng, Meng Liu, Li Li, Liqi Feng, Baili Song, Han Han-Zhang, Qingyang Ma, Lei Qian

**Affiliations:** a Department of Endocrinology and Metabolism, Peking University People's Hospital, Beijing, China; b The First Affiliated Hospital and Clinical Medicine College, Henan University of Science and Technology, Luoyang, China; c Innovent Biologics, Suzhou, China

## Abstract

**Background:**

IBI362 (LY3305677) is a novel weekly-dose glucagon-like peptide-1 and glucagon receptor dual agonist being developed for the treatment of obesity and type 2 diabetes. The aim of this randomised, placebo-controlled, multiple ascending dose phase 1b study was to evaluate the safety, tolerability, pharmacokinetics and efficacy of IBI362 in Chinese adults with overweight or obesity.

**Methods:**

This study enrolled adults with overweight (body mass index [BMI]≥24 kg/m^2^) accompanied by hyperphagia and/or at least one comorbidity or obesity (BMI≥28 kg/m^2^) from six study centres in China. Eligible participants were randomised 2:1 to receive once-weekly subcutaneous injection of IBI362 or placebo in each of the three ascending dose cohorts for 12 weeks with additional 8 weeks of safety follow-up. The dose-escalation regimens were: 3.0 mg cohort (1.0 mg weeks 1–4; 2.0 mg weeks 5–8; 3.0 mg weeks 9–12); 4.5 mg cohort (1.5 mg weeks 1–4; 3.0 mg weeks 5–8; 4.5 mg weeks 9–12); 6.0 mg cohort (2.0 mg weeks 1–4; 4.0 mg weeks 5–8; 6.0 mg weeks 9–12). The participants, investigators and study site personnel involved in treating and assessing participants within each cohort were masked to treatment allocation. The primary endpoints were safety and tolerability of IBI362. This study is registered with ClinicalTrials.gov, number NCT04440345.

**Findings:**

Between June 15^th^, 2020 and January 15^th^, 2021, 12 participants were enrolled and randomised in each cohort. Throughout the study, no participant discontinued the treatment due to safety reason and no serious adverse event was reported. Gastrointestinal adverse events and decreased appetite were the most common adverse events and mostly mild in severity. Three participants receiving IBI362 reported mild and asymptomatic cardiac disorders revealed by electrocardiogram. Estimated percent changes in mean body weight from baseline to week 12 were −4.81% (95%CI −6.61 to −3.02), −6.40% (−8.23 to −4.58) and −6.05% (−7.91 to −4.18) for participants receiving IBI362 in the 3.0 mg, 4.5 mg and 6.0 mg cohort, respectively, compared with 0.60% (−0.86 to 2.07) for those receiving placebo.

**Interpretation:**

IBI362 was well tolerated and showed a body weight-lowering effect in Chinese adults with overweight or obesity.

**Funding:**

Innovent Biologics.

**Research in context**

**Evidence before this study**

A PubMed search on April 21^st^, 2021, with the terms “glucagon-like peptide-1″, [AND] “glucagon receptor”, [AND] “dual agonist”, in the Title or Abstract yielded 21 results. Two daily dose GLP-1 and glucagon receptor dual agonists, MEDI0382 and SAR425899, had been evaluated in overweight or obese patients with type 2 diabetes. Two studies evaluated the efficacy and safety of JNJ-64565111, a weekly-dose GLP-1 and glucagon receptor dual agonist, in individuals with obesity without type 2 diabetes and individuals with type 2 diabetes and obesity.

**Added value of this study**

This study is the first to report the safety, tolerability, pharmacokinetics and primary efficacy of a weekly-dose GLP-1 and glucagon receptor dual agonist in Chinese adults with overweight or obesity. IBI362 demonstrated favourable safety profile and achieved superior body weight-lowering effect, together with improvements in multiple metabolic parameters.

**Implication of all the available evidence**

The efficacy of several daily dose GLP-1 and glucagon receptor dual agonists may currently be comparable but not superior to that of GLP-1 receptor agonists. IBI362 holds great potential for the treatment of obesity and other metabolic disorders. An ongoing phase 1b trial adopting escalation regimens with higher doses and a phase 2 trial will further evaluate the efficacy and safety of IBI362 in Chinese adults with overweight or obesity.

Alt-text: Unlabelled box

## 1 Introduction

Obesity represents a major public health problem in many countries and a global epidemic [1]. According to the World Health Organization (WHO), in 2016, more than 1.9 billion adults aged 18 years and older were overweight, and of those, over 650 million had obesity [2]. In China, the prevalence of general obesity increased 3-fold and that of abdominal obesity by more than 50% from 2004 to 2014 [3].

Obesity is a risk factor for a number of chronic diseases including but not limited to type 2 diabetes, hypertension, dyslipidaemia, coronary heart disease, stroke, musculoskeletal disorders and several cancers [2]. Although a weight reduction of 5–10% conveys multiple cardiovascular and metabolic benefits, this goal is difficult to achieve and possibly more difficult to maintain with lifestyle intervention and traditional anti-obesity medication [4,5].

Gastrointestinal peptides have emerged as a promising avenue to the treatment of obesity and metabolic disorders [6,7]. With the success of several approved glucagon-like peptide-1 (GLP-1) receptor agonists, single molecules with balanced activities at multiple gastrointestinal hormone receptors are expected to achieve addictive or even synergistic therapeutic outcomes with improved safety profiles [8,9]. Oxyntomodulin (OXM) is a gut hormone that activates both the GLP-1 receptor and the glucagon receptor [10]. The anorectic effect and increased energy expenditure of OXM are mediated by activation of GLP-1 receptor and glucagon receptor, respectively [10,11]. When administered exogenously, OXM can improve glucose tolerance and result in weight loss, making GLP-1 receptor and glucagon receptor dual agonists a new promising treatment option for patients with diabetes and/or obesity. It may have the potential to achieve superior weight loss and glucose-lowering effect compared with those of GLP-1 receptor agonists [10].

IBI362 is a long-acting synthetic peptide analogue of mammalian OXM, with a fatty-acyl moiety to extend the half-life [12]. IBI362 potently bound to human and mouse GLP-1 receptors and glucagon receptors in vitro. IBI362 improved glucose control in mice. Of note, IBI362 decreased body weight in both *Gcgr* knockout (KO) and *Glp1r* KO mice, indicating that activation of both receptors contributed to greater weight loss. Moreover, IBI362, but not semaglutide, a GLP-1 receptor agonist, increased energy expenditure as compared with vehicle [13]. Furthermore, a first-in-human single ascending dose study demonstrated the safety, glycaemic improvement and weight loss of IBI362 in healthy subjects [12].

This randomised, placebo-controlled, multiple ascending dose phase 1b trial evaluated the safety, tolerability, pharmacokinetics and efficacy of IBI362 in Chinese adults with overweight or obesity.

## 2 Methods

### 2.1 Study design

This randomised, placebo-controlled, dose-escalation, multiple ascending dose study was designed to explore the optimal dosing regimen and assess the safety, tolerability, pharmacokinetics and efficacy of IBI362 in Chinese adults with overweight or obesity. Participants were enrolled from 6 study centres in China, including Peking University People's Hospital (reference number for the ethics committee, 2020PHA015-001), Henan University of Science and Technology the First Affiliated Hospital (2020-0047), Shanxi Medical University the First Hospital (2020-42), Bengbu Medical College the First Affiliated Hospital (2020-055), Pingxiang People's Hospital (2020D0.0-F01) and Tonghua Central Hospital (YW-2020-009). The study was done in accordance with local laws, the International Conference on harmonisation Good Clinical Practice guidelines, and the ethical principles outlined in the Declaration of Helsinki. Respective ethics committees reviewed the study protocol and approved the study. This study is registered with ClinicalTrials.gov, number NCT04440345.

### 2.2 Participants

Adults with overweight (body mass index [BMI]≥24 kg/m^2^) accompanied by hyperphagia and/or at least one comorbidity (pre-diabetes, hypertension, dyslipidemia, fatty liver, weight-bearing arthralgia or dyspnea, obstructive sleep apnea syndrome caused by obesity) or obesity (body mass index≥28 kg/m^2^) and with less than 5% weight loss by diet and exercise 12 weeks or longer prior to screening were eligible for this study. The key exclusion criteria were concurrent or previous use of GLP-1 receptor agonists, use of weight-loss or anti-obesity agents three months prior to screening (for the full list of eligibility criteria, see the appendix). All participants provided written informed consent before study entry.

### 2.3 Randomisation and masking

An interactive web-response system generated identification numbers that were used to randomly assign eligible participants, who were randomised 2:1 to receive IBI362 or placebo in each cohort. Randomisation list was generated by an in-house statistician who was not involved in the clinical operations of the study. The study drugs and placebo were identically labelled and indistinguishable in appearance. As such, the participants, investigators, study site personnel involved in treating and assessing participants and sponsor personnel in each cohort were masked to treatment allocation.

### 2.4 Procedures

The study included a 3-week screening period, a 12-week treatment period and an 8-week safety follow-up period. During the 12-week treatment period, the dose of IBI362 or placebo was escalated within each cohort according to one of the three dose-escalation schedules (Fig. S1). The dose-escalation regimen for the 3.0 mg cohort was 1.0 mg for 4 weeks, followed by 2.0 mg for 4 weeks and then 3.0 mg for the final 4 weeks. The dose-escalation regimen for the 4.5 mg cohort was 1.5 mg for 4 weeks, followed by 3.0 mg for 4 weeks and then 4.5 mg for the final 4 weeks. The dose-escalation regimen for the 6.0 mg cohort was 2.0 mg for 4 weeks, followed by 4.0 mg for 4 weeks and then 6.0 mg for the final 4 weeks. The 6.0 mg cohort started dosing after tolerability evaluation of 1.5 mg dosing in the 4.5 mg cohort. The internal unblinded safety monitoring group was responsible for the safety monitoring. All treatments were subcutaneously administered once a week. Investigators and authorized study personnel in each centre recorded the study data into the electronic case report forms (eCRFs), which were reviewed and validated by clinical research associates (CRAs). The data in the eCRFs were submitted to the electronic data capture (EDC) database. HbA_1c_, fasting plasma glucose and fasting insulin tests were performed in a central lab (Wuxi AppTec Inc.).

### 2.5 Outcomes

The primary endpoints of the study were safety and tolerability of IBI362. Adverse events (AEs) were categorised according to the Medical Dictionary for Regulatory Activities system organ class and preferred terms. Treatment emergent adverse events (TEAEs) and serious adverse events were monitored in all participants throughout the treatment period and the safety follow-up period. The severity of the AEs (mild, moderate or severe) and the association between an AE and the study drug was assessed by investigators based on pre-specified criteria.

The secondary pharmacodynamic endpoints included changes from baseline (pre-dose on day 0) to week 12 in fasting plasma glucose and fasting insulin levels. The secondary efficacy endpoints included changes from baseline to week 12 in body weight, waist circumference, waist-to-hip ratio, BMI, blood pressure, heart rate, lipids and HbA_1c_ levels. Post-hoc comparison of changes from baseline to week 12 in serum uric acid levels was done between participants receiving IBI362 in each cohort and those receiving placebo.

Pharmacokinetic parameters were assessed on pre-defined visits in all participants who received IBI362. These parameters included maximum observed plasma concentration (C_max_), time at which C_max_ was observed (T_max_), terminal elimination half-life (T_1/2_), and AUC from time zero to 168 h after the first dose (AUC_0–168__h_). Standard non-compartmental pharmacokinetics methods were used to analyze IBI362 plasma concentration data using Pkanalix 2020R1 (Lixoft, Antony, France). The values for C_max_ and T_max_ were taken directly from the observed plasma concentration data. The terminal-phase disposition rate constant (λz) was estimated using a log-linear regression of the concentration data in the terminal disposition phase, and T_1/2_ was estimated as ln(2)/λz. Immunogenicity was assessed by measures of anti-drug antibody-positive responses.

### 2.6 Statistical analysis

The sample size of 12 participants per cohort was empirically determined to provide adequate safety, tolerability, pharmacokinetics and pharmacodynamic data while minimising the number of participants. No formal analyses between cohorts were planned but descriptive results were shown. No formal hypothesis testing was planned. Participants receiving placebo were pooled for analysis.

Safety analyses were done in the safety population (defined as all participants who received at least one dose of the study treatment). Pharmacokinetic analyses were done in the pharmacokinetic population (defined as all participants who received at least one dose of IBI362 and had at least one valid plasma concentration assessment). Pharmacodynamic analyses were done in the pharmacodynamic population (defined as all participants who received at least one dose of the study treatment and had baseline and at least one valid assessment). Efficacy analyses were done in the efficacy population (defined as all participants who received at least one dose of the study treatment and had at least one post-baseline assessment).

We used mixed effect model for repeated measures (MMRM) for post-baseline repeated measures to analyse continuous efficacy and pharmacodynamic endpoints. The MMRM included the corresponding baseline value, treatment, visit and treatment-by-visit as fixed effects and participant as a random effect. Mean change from baseline or mean percent change from baseline were analysed using a restricted maximum likelihood (REML)-based repeated measures approach in combination with the Newton Raphson Algorithm. The fix effect of visit was fitted as a repeated effect and an unstructured covariance matrix was used. In case of non-converge, matrix like Variance Components and Compound Symmetry will be tested in a subsequent order until model-convergence is achieved. The between-subject degree of freedom was used in estimating fixed effects. No missing data imputation was conducted. Least squares mean estimates of treatment effect at week 12 as well as treatment difference versus placebo alongside with two-sided 95% confidence intervals were provided for key efficacy and pharmacodynamic endpoints. Significance test for body weight was based on least squares means using a two-sided α of 0.05.

HbA_1c_ was measured only at baseline and week 12. Least squares mean estimates for HbA_1c_ were computed from an Analysis of Covariance (ANCOVA) model with baseline HbA_1c_ value and treatment as covariates. Last observation carried forward (LOCF) was used for imputing missing post-baseline measure. Least squares mean estimates of treatment effect at week 12 alongside with two-sided 95% confidence intervals were provided.

Post-hoc analysis of serum uric acid was performed using MMRM model described above.

All statistical analyses were done using SAS version 9.4.

### 2.7 Role of the funding source

This study was sponsored by Innovent Biologics. The funding was used for study design, data collection, data analysis, and data interpretation. All authors verified that this study was done according to the protocol and was attested for data accuracy and completeness. All authors had full access to all the data in the study, contributed to writing and reviewing of the manuscript, and approved the final submitted version. The corresponding author had final responsibility for the decision to submit for publication.

## 3 Results

### 3.1 Participants

**Fig. 1:**
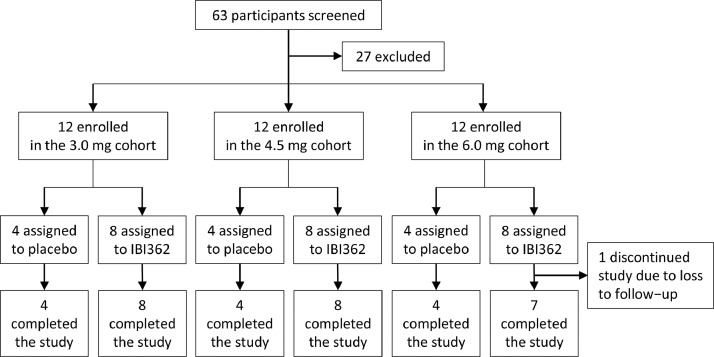
Trial profile.

Between June 15^th^, 2020 and January 15^th^, 2021, 63 individuals were screened for eligibility, of whom 36 were enrolled. Twelve participants were enrolled in each dose cohort and randomised 2:1 to receive IBI362 or placebo. Demographic and baseline characteristics were essentially balanced between different treatment cohorts (Table 1 and S1). One participant dropped out at week 6 due to loss to follow-up. The remaining 35 participants completed the study (Fig. 1). All participants (*n* = 36) were included in the safety, pharmacodynamic and efficacy analyses. All participants receiving IBI362 (*n* = 24) were included in the pharmacokinetic analyses.

**Table 1:** Participant demographics and baseline characteristics (safety population).

	3.0 mg cohort (*n* = 12)		4.5 mg cohort (*n* = 12)		6.0 mg cohort (*n* = 12)		Pooled placebo (*n* = 12)
	IBI362 (*n* = 8)	placebo (*n* = 4)	IBI362 (*n* = 8)	placebo (*n* = 4)	IBI362 (*n* = 8)	placebo (*n* = 4)	
Sex							
Male	3 (37.5%)	1 (25.0%)	6 (75.0%)	2 (50.0%)	5 (62.5%)	1 (25.0%)	4 (33.3%)
Female	5 (62.5%)	3 (75.0%)	2 (25.0%)	2 (50.0%)	3 (37.5%)	3 (75.0%)	8 (66.7%)
Race							
Asian	8 (100.0%)	4 (100.0%)	8 (100.0%)	4 (100.0%)	8 (100.0%)	4 (100.0%)	12 (100.0%)
Age (years)	29.5 (24.0–45.0)	39.5 (31.0–43.5)	31.0 (23.5–33.0)	37.0 (32.5–43.0)	40.0 (23.0–51.5)	32.0 (28.5–34.5)	35.0 (30.0–40.0)
BMI (kg/m²)	29.3 (2.2)	30.6 (1.2)	32.4 (3.4)	29.1 (3.7)	31.7 (2.0)	29.0 (5.1)	29.6 (3.4)
24≤BMI<28 (overweight)	2 (25.0%)	0 (0)	1 (12.5%)	1 (25.0%)	0 (0)	3 (75.0%)	4 (33.3%)
BMI>28 (obesity)	6 (75.0%)	4 (100.0%)	7 (87.5%)	3 (75.0%)	8 (100%)	1 (25.0%)	8 (66.7%)
Body weight (kg)	78.3 (13.7)	81.7 (3.3)	93.1 (9.9)	82.7 (13.7)	87.4 (8.8)	76.5 (21.5)	80.3 (13.7)
Waist circumference (cm)	93.4 (8.9)	95.1 (2.0)	105.1 (8.0)	98.1 (10.5)	104.8 (10.2)	90.1 (14.9)	94.4 (10.2)
Blood pressure (mm Hg)							
Systolic	118.5 (10.5)	118.3 (10.5)	122.0 (11.3)	116.5 (8.2)	124.1 (6.9)	109.5 (10.0)	114.8 (9.5)
Diastolic	79.3 (9.7)	82.3 (14.6)	80.8 (7.4)	81.3 (7.9)	81.3 (7.3)	70.5 (6.1)	78.0 (10.8)
HbA_1c_ (%)	5.3 (0.6)	5.4 (0.2)	5.2 (0.2)	5.2 (0.3)	5.3 (0.3)	5.1 (0.3)	5.2 (0.3)
Fasting plasma glucose (mmol/L)	5.5 (5.4–5.7)	5.5 (5.4–5.6)	5.4 (5.1–5.5)	5.6 (5.0–5.9)	5.1 (5.0–5.8)	5.7 (5.4–5.9)	5.5 (5.3–5.8)
Fasting insulin (µU/mL)	13.9 (11.1–20.3)	12.9 (10.1–25.6)	17.0 (11.9–24.7)	10.4 (8.3–15.5)	16.1 (14.1–24.4)	13.9 (10.7–53.1)	11.3 (10.1–18.1)
Total cholesterol (mmol/L)	4.8 (4.4–6.0)	4.7 (3.5–6.4)	4.9 (4.4–5.1)	4.8 (4.1–5.3)	4.2 (3.5–4.8)	4.4 (3.8–4.7)	4.6 (3.6–5.3)
LDL cholesterol (mmol/L)	3.0 (2.2–3.8)	2.7 (1.6–3.9)	3.2 (2.9–3.7)	2.5 (1.9–3.5)	2.5 (2.3–2.7)	3.1 (2.5–3.3)	3.0 (2.0–3.3)
Triglycerides (mmol/L)	1.2 (1.1–1.8)	1.9 (1.4–3.1)	1.6 (1.3–1.8)	2.1 (1.0–3.7)	1.5 (1.0–2.7)	1.1 (0.8–2.0)	1.5 (1.0–2.8)

Data are presented as mean (SD), median (interquartile range) or n (%). BMI = body mass index. HbA_1c_ = glycated hemoglobin A1c. LDL = low density lipoprotein.

### 3.2 Safety

IBI362 was generally well tolerated and safe. No participant discontinued the study due to safety reason. No hypoglycaemia event or serious adverse event occurred. During the dose escalation period, no dose adjustment of the study drug was made.

Overall, 19 participants (79.2%) receiving IBI362 and seven (58.3%) receiving placebo experienced at least one TEAE, with gastrointestinal adverse events (GI AEs) and decreased appetite being the most common TEAEs. Decreased appetite was reported in seven participants (29.2%) receiving IBI362 and two (16.7%) receiving placebo. Diarrhea was reported in six participants (25.0%) receiving IBI362 and one (8.3%) receiving placebo. Nausea was reported in four participants (16.7%) receiving IBI362 and one (8.3%) receiving placebo (Table 2 and S2). Except that one participant receiving IBI362 in the 3.0 mg cohort experienced moderate gastrointestinal inflammation, which was unrelated to the treatment as judged by the investigator, and one receiving IBI362 in the 6.0 mg cohort experienced moderate but transient vomiting and nausea, all other GI AEs were mild in severity. GI AEs and decreased appetite occurred most frequently in participants receiving IBI362 in the 4.5 mg cohort, predominantly within 3–4 days after each dose (Fig. S2).

**Table 2:** Overview of treatment emergent adverse events (safety population).

n (%)	3.0 mg cohort (*n* = 12)		4.5 mg cohort (*n* = 12)		6.0 mg cohort (*n* = 12)		Pooled placebo (*n* = 12)
	IBI362 (*n* = 8)	placebo (*n* = 4)	IBI362 (*n* = 8)	placebo (*n* = 4)	IBI362 (*n* = 8)	placebo (*n* = 4)	
**All TEAE**							
TEAE	6 (75.0)	2 (50.0)	7 (87.5)	2 (50.0)	6 (75.0)	3 (75.0)	7 (58.3)
Treatment-related AE	3 (37.5)	1 (25.0)	7 (87.5)	1 (25.0)	5 (62.5)	1 (25.0)	3 (58.3)
Serious adverse event	0	0	0	0	0	0	0
Hypoglycaemia event	0	0	0	0	0	0	0
Gastrointestinal AE	3 (37.5)	1 (25.0)	5 (62.5)	1 (25.0)	4 (50.0)	1 (25.0)	3 (25.0)
Moderate	1 (12.5)	0	0	0	1 (12.5)	0	0
Mild	2 (25.0)	1 (25.0)	5 (62.5)	1 (25.0)	3 (37.5)	1 (25.0)	3 (25.0)
**Gastrointestinal AE**							
Constipation	1 (12.5)	0	0	0	0	1 (25.0)	1 (8.3)
Vomiting	1 (12.5)	0	0	0	1 (12.5)	0	0
Nausea	0	0	3 (37.5)	1 (25.0)	1 (12.5)	0	1 (8.3)
Dyspepsia	0	0	0	0	1 (12.5)	0	0
Gastrointestinal inflammation	1 (12.5)	0	0	0	0	0	0
Diarrhea	0	0	3 (37.5)	1 (25.0)	3 (37.5)	0	1 (8.3)
Abdominal pain	0	1 (25.0)	2 (25.0)	0	0	0	1 (8.3)
Abdominal distension	1 (12.5)	0	0	0	0	0	0
**Other events of special interest**							
Decreased appetite	1 (12.5)	0	5 (62.5)	1 (25.0)	1 (12.5)	1 (25.0)	2 (16.7)
Hiccups	0	0	0	0	1 (12.5)	0	0
Atrioventricular block first degree	1 (12.5)	0	0	0	0	0	0
Myocardial ischemia	0	0	1 (12.5)	0	0	0	0
Sinus tachycardia	0	0	1 (12.5)	0	0	0	0
Urticaria	1 (12.5)	1 (25.0)	0	0	0	0	1 (8.3)

Data are presented as n (%). TEAE = treatment emergent adverse event. AE = adverse event.

Heart rate increase was observed in all treatment groups. The magnitude of heart rate increase was mild and similar in participants receiving different doses of IBI362 and placebo for the first 6 weeks. The heart rate increase was more prominent in the latter part of the dose escalation period especially in participants receiving IBI362 in the 6.0 mg cohort but tended to decline from week 9 towards the end of treatment (Fig. S3).

Amylase, lipase and calcitonin levels increased in all IBI362 treatment groups but remained mostly within the normal reference range (Fig S4). No investigator suspected pancreatitis was reported. There were no reports of thyroid tumours, neoplasms or C-cell hyperplasia events.

One participant receiving IBI362 in the 3.0 mg cohort reported two episodes of atrioventricular block first degree. One participant receiving IBI362 in the 4.5 mg cohort reported myocardial ischemia and another one in the same cohort reported sinus tachycardia (Table 2 and S2). No cardiac AE was reported in participants receiving IBI362 in the 6.0 mg cohort. All cardiac disorders were related to IBI362 as assessed by the investigators, albeit mild, asymptomatic, and without dose adjustment or discontinuation of the study drug.

### 3.3 Pharmacokinetics

Table 3 showed non-compartmental pharmacokinetic parameters following subcutaneous administration of the first doses. IBI362 demonstrated slow absorption with peak concentrations (C_max_) achieved ranging from 11.8 h to 120 h, and the median T_max_ is 72 h. Once past the T_max_, IBI362 concentrations declined slowly over several weeks with half-life (T_1/2_) ranging from 150.9 h to 403.5 h (6.3 days to 16.8 days). One participant receiving IBI362 in the 3.0 mg cohort and one receiving IBI362 in the 4.5 mg cohort developed anti-IBI362 antibody after receiving IBI362.

**Table 3:** Pharmacokinetic parameters (pharmacokinetic population).

	IBI362 1.0–2.0–3.0 mg (*n* = 8)	IBI362 1.5–3.0–4.5 mg (*n* = 8)	IBI362 2.0–4.0–6.0 mg (*n* = 8)
C_max_ (ng/mL)	82.4 (16.2)	99.6 (31)	134.1 (25.9)
T_max_ (h)	71.9 (48–95.8)	84.5 (11.8–120)	71.9 (48.1–95.9)
AUC_0–168__h_ (ng•h/mL)	12,548.7 (18.1)	15,569.2 (30.6)	19,188.2 (27.7)

C_max_ and AUC are presented as geometric mean (CV%). T_max_ is presented as median (range). AUC_0–168_ *_h_* = area under the concentration versus time curve from time zero to 168 h; C_max_ = maximum observed drug concentration; CV% = coefficient of variation; T_max_ = time of C_max_.

### 3.4 Efficacy endpoints

**Fig. 2:**
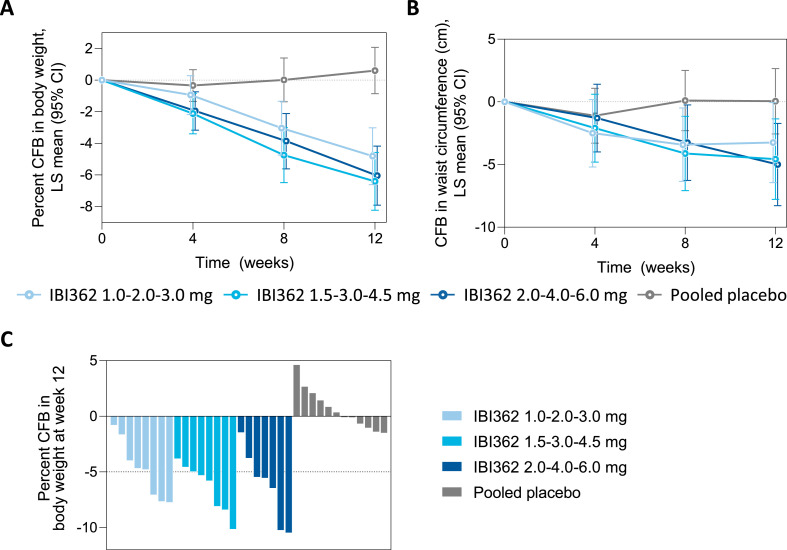
**Changes in body weight and waist circumference.** A. Percent changes in body weight from baseline to week 12. B. Changes in waist circumference from baseline to week 12. C. Percent changes in body weight from baseline to week 12 for each participant. CFB = change from baseline. CI = confidence interval. LS = least squares.

At week 12, all doses of IBI362 reduced body weight, BMI and waist circumference from baseline (Fig. 2, A and B, Table 4). Percent changes in mean body weight from baseline to week 12 were −4.81% (95%CI −6.61 to −3.02), −6.40% (−8.23 to −4.58) and −6.05% (−7.91 to −4.18) for participants receiving IBI362 in the 3.0 mg, 4.5 mg and 6.0 mg cohort, respectively, compared with 0.60% (−0.86 to 2.07) for those receiving placebo. Estimated treatment difference versus placebo were −5.42% (95% CI −7.71 to −3.12) for participants receiving IBI362 in the 3.0 mg cohort, −7.01% (−9.39 to −4.63) for those receiving IBI362 in the 4.5 mg cohort, and −6.65% (−9.03 to −4.27) for those receiving IBI362 in the 6.0 mg cohort (*p*<0.0001 for all comparisons). Thirteen participants (54.2%) treated with IBI362 achieved ≥5% weight loss from baseline at week 12 (versus 0% with placebo) (Fig. 2C). Changes in mean BMI from baseline to week 12 ranged from −1.44 kg/m² to −2.00 kg/m² for IBI362 (versus 0.14 kg/m² for placebo). Changes in mean waist circumference from baseline to week 12 ranged from −3.25 cm to −5.00 cm for IBI362 (versus 0.05 cm for placebo) (Table 4).

**Table 4:** Key secondary endpoints (pharmacodynamic/efficacy population).

		IBI362 1.0–2.0–3.0 mg(*n* = 8)	IBI362 1.5–3.0–4.5 mg(*n* = 8)	IBI362 2.0–4.0–6.0 mg(*n* = 7)	Pooled placebo(*n* = 12)
Body weight	CFB (kg)	−3.80 (−5.16 to −2.44)	−5.77 (−7.16 to −4.38)	−5.12 (−6.53 to −3.71)	0.37 (−0.73 to 1.48)
	Diff. to placebo (kg)	−4.18 (−5.91 to −2.45)	−6.14 (−7.95 to −4.34)	−5.49 (−7.29 to −3.69)	
	p value	< 0.0001	< 0.0001	< 0.0001	
	Percent CFB (%)	−4.81 (−6.61 to −3.02)	−6.40 (−8.23 to −4.58)	−6.05 (−7.91 to −4.18)	0.60 (−0.86 to 2.07)
	Diff. to placebo (%)	−5.42 (−7.71 to −3.12)	−7.01 (−9.39 to −4.63)	−6.65 (−9.03 to −4.27)	
	p value	< 0.0001	< 0.0001	< 0.0001	
BMI (kg/m²)	CFB	−1.44 (−1.96 to −0.91)	−2.00 (−2.53 to −1.47)	−1.88 (−2.43 to −1.33)	0.14 (−0.29 to 0.57)
Waist circumference (cm)	CFB	−3.25 (−6.44 to −0.06)	−4.57 (−7.78 to −1.36)	−5.00 (−8.28 to −1.72)	0.05 (−2.56 to 2.65)
Diastolic blood pressure (mm Hg)	CFB	−8.48 (−13.00 to −3.96)	−5.76 (−10.28 to −1.23)	−2.73 (−7.52 to 2.06)	1.89 (−1.81 to 5.60)
Systolic blood pressure (mm Hg)	CFB	−8.26 (−14.77 to −1.76)	−5.80 (−12.34 to 0.73)	−5.59 (−12.56 to 1.38)	2.02 (−3.40 to 7.44)
HbA1c (%)	CFB	−0.38 (−0.51 to −0.26)	−0.24 (−0.36 to −0.11)	−0.23 (−0.35 to −0.1)	−0.03 (−0.14 to 0.07)
Fasting plasma glucose (mmol/L)	CFB	−0.30 (−0.58 to −0.02)	−0.11 (−0.39 to 0.17)	−0.13 (−0.43 to 0.16)	0.16 (−0.07 to 0.38)
Fasting insulin (µU/mL)	CFB	−3.58 (−10.15 to 2.99)	−6.14 (−12.69 to 0.40)	−0.42 (−7.37 to 6.54)	1.60 (−3.74 to 6.95)
Total cholesterol (mmol/L)	CFB	−0.74 (−1.17 to −0.32)	−0.68 (−1.10 to −0.26)	−0.81 (−1.26 to −0.36)	−0.09 (−0.44 to 0.25)
LDL cholesterol (mmol/L)	CFB	−0.11 (−0.44 to 0.21)	−0.25 (−0.58 to 0.09)	−0.34 (−0.68 to 0.01)	0 (−0.27 to 0.27)
Triglycerides (mmol/L)	CFB	−0.44 (−0.80 to −0.08)	−0.55 (−0.90 to −0.19)	−0.74 (−1.11 to −0.36)	−0.03 (−0.32 to 0.26)

Data are presented as LS mean (95% CI). P values are for treatment difference versus placebo (unadjusted for multiple comparisons). LS mean estimates were calculated from a MMRM model with corresponding baseline measures, visit, treatment and treatment by visit as fixed effects and unstructured covariance. LS means estimates of HbA_1c_ were calculated from an ANCOVA model with baseline values and treatment as covariates and LOCF for missing data imputation.

BMI = body mass index. HbA_1c_ = glycated haemoglobin A1c. CFB = change from baseline. Diff = difference. LDL = low density lipoprotein. LS = least squares. CI = confidence interval. MMRM = mixed effect model for repeated measures. ANCOVA = Analysis of Covariance. LOCF = last observation carried forward.

At week 12, more pronounced reductions in blood pressure, HbA_1c_, fasting plasma glucose, fasting insulin, cholesterol and triglycerides levels were observed in participants receiving IBI362, compared to placebo (Table 4).

Serum alanine aminotransferase (ALT) and aspartate aminotransferase (AST) levels also decreased from baseline in participants receiving IBI362 in all three dose cohorts. Decreases in participants receiving IBI362 in the 3.0 mg and 4.5 mg cohorts were greater than in those receiving placebo (Fig. S6).

**Fig. 3:**
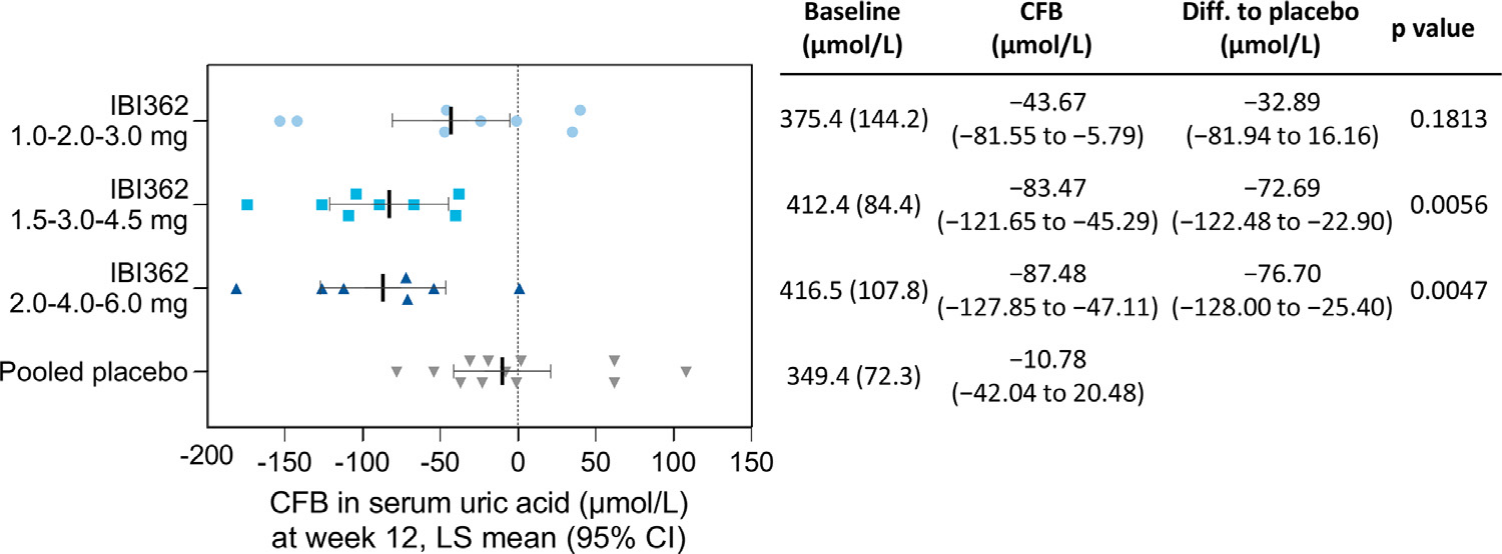
**Post-hoc analysis of changes in uric acid levels from baseline to week 12.** Data are LS mean (95% CI) or mean (SD). *P* values are for treatment difference versus placebo (unadjusted for multiple comparisons). LS mean estimates were calculated from a MMRM model with corresponding baseline measures, visit, treatment and treatment by visit as fixed effects and unstructured covariance. Each dot represents CFB of one participant. CFB = change from baseline. Diff = difference. LS = least squares. CI = confidence interval. MMRM = mixed effect model for repeated measures. SD = standard deviation.

Post-hoc analysis revealed that all doses of IBI362 reduced concentration of serum uric acid from baseline to week 12 (Fig. 3). Changes in mean serum uric acid levels from baseline to week 12 were −43.67 µmol/L (95%CI −81.55 to −5.79), −83.47 µmol/L (−121.65 to −45.29) and −87.48 µmol/L (−127.85 to −47.11) for participants receiving IBI362 in the 3.0 mg, 4.5 mg and 6.0 mg cohort, respectively, compared with −10.78 µmol/L (−42.04 to 20.48) for those receiving placebo. Participants receiving IBI362 in the 4.5 mg and 6.0 mg cohorts achieved statistically significant reductions in serum uric acid levels versus placebo, with estimated treatment difference of −72.69 µmol/L (95% CI −122.48 to −22.90 *p* = 0.0056) and −76.70 µmol/L (−128 to −25.40 *p* = 0.0047), respectively. Concurrent reductions in serum uric acid levels and body weight were observed in most participants receiving IBI362 (Fig. S5).

## 4 Discussion

This 12-week randomised, placebo-controlled phase 1b study evaluated the safety, tolerability, pharmacokinetics and efficacy of IBI362, a novel weekly-dose GLP-1 and glucagon receptor dual agonist, in Chinese adults with overweight or obesity. IBI362 was well tolerated and showed a safety profile similar to other GLP-1 receptor agonists and GLP-1 based receptor co-agonists. At week 12, 10 (41.7%) participants receiving IBI362 (versus 0 receiving placebo) experienced coincident decrease in body weight, low density lipoprotein (LDL) cholesterol levels, triglycerides levels, blood pressure and serum uric acid levels (data not shown). IBI362 demonstrated great potential for weight loss and improvements in multiple metabolic parameters in adults with overweight or obesity.

The three dose escalation regimens in this study were well tolerated. Except that one participant dropped out due to loss to follow-up, all randomised participants completed the study without dose adjustment. The most common TEAEs were GI AEs and decreased appetite, among which most were mild or moderate in severity. GI AEs and decreased appetite appeared more frequently reported in the first two dose levels of each dose escalation regimen, suggesting tolerance may be induced prior to the exposure to higher doses. Due to small sample size, dose-dependent AE occurrence was not observed in this study. GI AEs and decreased appetite occurred most frequently in participants receiving IBI362 in the 4.5 mg cohort and most events were reported within 3–4 days following each dose, which was consistent with T_max_.

Slight increase in heart rate is associated with the use of GLP-1 receptor agonists. Although evidence suggested OXM increased intrinsic heart rate through the glucagon receptor in mice [14], heart rate increase observed in the latter part of the dose escalation period in participants with IBI362 was mild and generally comparable with other GLP-1 based receptor agonists and co-agonists [15], [16], [17].

Cardiac events are AEs of special clinical interest for GLP-1 based receptor agonists and co-agonists. In total, 4 episodes of asymptomatic cardiac AEs, all revealed by electrocardiogram, occurred in participants receiving IBI362 in the 3.0 mg and 4.5 mg cohort. No cardiac event was reported in participants receiving IBI362 in the 6.0 mg cohort with a more prominent heart rate increase.

Pharmacokinetic profile supported the weekly subcutaneous injection of IBI362, with half-life extended by the addition of a fatty acyl side chain to the OXM analogue. The long-acting pharmacokinetic characteristics confer both patient convenience and pharmacodynamic benefits, as high and sustained concentrations of selective GLP-1 receptor agonists have enhanced clinical efficacy [18,19].

For GLP-1 and glucagon receptor dual agonists, the weight loss is expected from both the inhibition of increased satiety and reduced dietary intake induced by GLP-1 receptor agonism, and increase in energy expenditure by glucagon receptor stimulation [20,21]. We observed an estimated mean reduction in body weight from 4.18 kg to 6.14 kg (from 5.42% to 7.01%) versus placebo at week 12 in participants treated with IBI362. The most prominent weight loss was achieved in participants receiving IBI362 in the 4.5 mg cohort, who were the youngest and with the highest baseline body weight. The efficacy of IBI362 in body weight loss is comparable to state-of-the-art GLP-1 based receptor agonists and co-agonists. In STEP 1 study, 2.4 mg of semaglutide once weekly plus lifestyle intervention achieved weight loss by around 6% at week 12 and a further reduction by 12.4% versus placebo at week 68 in participant with obesity or overweight[22]. In SURPASS-2 trial, weekly injection of tirzepatide, a GLP-1 and GIP receptor dual agonist, achieved mean weight loss of about 5.5% at week 12 in adults with type 2 diabetes[23]. With the body weight loss achieved by IBI362 at week 12 and the body weight reduction trend over 12-week treatment period (Fig. 2), we believe that a greater magnitude of weight loss will be achieved with extended period of time and effect of different dose regimens should be evaluated thereafter.

Despite the weight loss effect of glucagon, its inherited stimulation of gluconeogenesis and glycogenolysis requires optimal balancing of GLP-1 and glucagon receptor activities for dual agonists. Cotadutide, a once-daily GLP-1 and glucagon receptor dual agonist, demonstrated optimal weight loss and glycaemic control in a 48-day phase 2a study in Japanese adults with type 2 diabetes [24]. JNJ-64565111, a once-weekly GLP-1 and glucagon receptor dual agonist, showed encouraging weight loss but no improvement in glycaemic parameters in adults with type 2 diabetes and obesity [25]. IBI362 has been designed to balance the activation of GLP-1 receptor and glucagon receptor in order to avoid the glucagon receptor-induced hyperglycaemia while maintaining the desired effects of HbA_1c_-lowering and body weight reduction. Evaluated in participants with essentially normal glycaemic parameters, IBI362 reduced HbA_1c_ levels by a mean of 0.20% to 0.35% and fasting plasma glucose levels by a mean of 0.27 mmol/L to 0.45 mmol/L at week 12 in participants receiving IBI362 in different dose cohorts. IBI362 is expected to convey glycaemic benefit in overweight or obesity patients with pre-diabetes or type 2 diabetes.

Apart from weight loss and glycaemic control, IBI362 elicited robust improvement in lipid profiles. The effects of glucagon on hepatic lipid metabolism has been well documented [26]. Glucagon receptor agonism may indirectly improve experimental components of non-alcoholic steatosis hepatitis via weight loss and through direct actions to reduce hepatic lipogenesis and augment mitochondrial oxidative capacity [27]. The reductions in total cholesterol, LDL cholesterol and triglycerides levels, together with a robust decrease in ALT and AST levels, confer comprehensive metabolic benefits to patients with obesity or type 2 diabetes and supported a further investigation and development of IBI362 in non-alcoholic steatosis hepatitis.

A remarkable and unexpected finding of this study was the statistically significant reduction in serum uric acid levels in participants receiving IBI362. BMI is strongly positively correlated with serum uric acid levels, and weight loss is a commonly recommended treatment for gout, a disease resulting from chronic elevation of serum uric acid [28], [29], [30]. Although GLP-1 receptor agonists promote weight loss, direct evidence regarding the relationship between GLP-1 receptor agonists and blood uric acid level is limited. One study demonstrated that 12-week administration of liraglutide in patients with type 2 diabetes did not reduce plasma uric acid levels compared with placebo [31]. Moreover, no plasma uric acid level change was reported with semeglutide and tirzepatide, both of which led to sharp body weight loss within 3–6 months. The effect of IBI362 on blood uric acid and underlying mechanisms warrant further investigation.

The limitations of the study include a limited number of participants of relatively young age, a short study duration, as well as the lack of central laboratory testing. Nevertheless, the favourable safety profile and preliminary efficacy of IBI362 in this study support IBI362 as a therapeutic agent for adults with overweight or obesity. Further trials have been planned to confirm these results in larger, multicentre, long-term studies with higher power, to evaluate the safety and efficacy of IBI362 in patients with obesity or type 2 diabetes.

In summary, this study demonstrated that IBI362 was well tolerated with an overall good safety profile in Chinese participants with overweight or obesity. IBI362 showed weight loss efficacy and metabolic improvement.

## Funding

This study was sponsored by Innovent Biologics.

## Contributors

LJ, LQ, HD and PA designed the study. LL, LF and BS analysed the data. LQ, HD, ML, PA, LL, LF, BS, HH-Z and QM interpreted the data. LJ, HJ and PA did the trial and collected the data. HH-Z and QM wrote the manuscript. All authors had full access to all the data in the study and had critically reviewed the manuscript and approved the final manuscript. All authors vouch for data accuracy and fidelity to the protocol.

## Data sharing statement

The data supporting the analyses contained in the manuscript will be made available upon reasonable written request to the corresponding author from researchers whose proposed use of the data for a specific purpose has been approved.

## Declaration of Competing Interest

LJ and HJ report personal fees from Innovent Biologics, during the conduct of the study. LQ, HD, ML, PA, LL, LF, BS, HH-Z and QM are employees of Innovent Biologics.

## Appendix Supplementary materials

Image, application 1
