# Supplementary material for: IBI362 (LY3305677), a weekly-dose GLP-1 and glucagon receptor dual agonist, in Chinese adults with overweight or obesity: A randomised, placebo-controlled, multiple ascending dose phase 1b study
Source: eClinicalMedicine. 2021 Aug 13;39:101088. doi: 10.1016/j.eclinm.2021.101088 (PMC8374649; doi:10.1016/j.eclinm.2021.101088)
Supplement: Supplementary file 1 [file mmc1.docx]

**Appendix**

**Supplementary text**

**Full eligibility criteria**

**Inclusion Criteria**

1. Aged 18 ~ 75 years (including both ends), male or female;
2. Obese: BMI ≥ 28.0 kg/m^2^; or overweight: 24 ≤ BMI < 28.0 kg/m^2^ accompanied by hyperphagia and/or at least one of the following comorbidity: i. Presence of one or several of prediabetes (impaired fasting glucose and/or impaired glucose tolerance), hypertension, dyslipidemia (see Appendix 4 for details of reference criteria), fatty liver (within 6 months prior to screening); ii. Combined with weight-bearing joint pain; iii. Dyspnea caused by obesity or obstructive sleep apnea syndrome;
3. Weight change of less than 5% controlled by simple diet and exercise for at least 12 weeks at screening;
4. Able to understand the procedures and methods of this study, willing to complete this trial in strict accordance with the clinical trial protocol, and voluntarily sign an informed consent form.

**Exclusion Criteria**

1. Patients suspected by the investigator to be allergic to the study drug or ingredients or patients with allergic constitution;
2. Use of any of the following medications or therapies prior to Screening:
3. Prior use of GLP-1 receptor (GLP-1R) agonists or GLP-1R/GCGR agonists;
4. Use of drugs that have an effect on body weight within 3 months before screening, including: systemic steroids (intravenous, oral, or intra-articular), metformin, SGLT2 inhibitors, thiazolidinediones (TZDs), tricyclic antidepressants, psychiatric or sedative drugs (such as promazine, amitriptyline, mirtazapine, paroxetine, phenelzine, chlorpromazine, thioridazine, chlorzapine, olanzapine, valproic acid, valproic acid derivatives, lithium);
5. Use of herbal medicines or health products that affect body weight within 3 months before screening;
6. Patients who have used or are currently using weight-loss drugs within 3 months before screening, such as: sibutramine hydrochloride, orlistat, phenylbutamine, phenylpropanolamine, chlorphenindole, finamine, bupropion, chlorkasaline, finamine/topiramate mixture, naltrexone/bupropion mixture, etc.;
7. Participation in other clinical trials within 3 months prior to screening (having received treatment with an experimental drug);
8. History or evidence of any of the following prior to Screening:
9. Patients diagnosed with diabetes by WHO 1999 criteria;
10. Fasting venous blood glucose ≥ 7.0 mmol/L or 2-hour venous blood glucose ≥ 11.1 mmol/L after a 75 g oral glucose tolerance test (OGTT) glucose load at screening (subjects with fasting blood glucose between 6.1 and 7.0 mmol/L at screening need to collect 2-hour venous blood glucose after an OGTT glucose load for confirmation);
11. Patients with previous or screening retinopathy;
12. Obesity caused by secondary diseases or drugs, including: elevated cortisol hormone (e.g., Cushing's syndrome), obesity caused by pituitary and hypothalamic injury, obesity caused by dose reduction/discontinuation of weight-loss drugs;
13. Previous bariatric surgery or acupuncture for weight loss within 1 year before screening;
14. History of depression; or history of severe mental illness, such as schizophrenia, bipolar disorder, etc.;
15. Uncontrolled hypertension at screening after at least 4 weeks of antihypertensive treatment, defined as systolic blood pressure > 140 mm Hg and/or diastolic blood pressure > 100 mm Hg;
16. Systolic blood pressure < 90 mm Hg and/or diastolic blood pressure < 50 mm Hg at screening;
17. History of malignancy at screening (except cured cutaneous basal cell carcinoma and cervical carcinoma in situ);
18. Cardiac-related diseases (such as angina pectoris, myocardial infarction, cardiomyopathy, acute and chronic heart failure, etc.) at screening;
19. Hemorrhagic or ischemic stroke or transient ischemic attack within 6 months prior to screening;
20. History of thyroid C-cell carcinoma, MEN (multiple endocrine neoplasia) 2A or 2B history or relevant family history at screening;
21. History of acute and chronic pancreatitis, gallbladder diseases, or pancreatic injury at screening;
22. Chronic gastrointestinal or systemic disease that may affect gastrointestinal motility at screening, or use of drugs that may alter gastrointestinal motility, appetite, or absorption within 3 months before screening;
23. Presence of limb deformity or mutilation, and unable to accurately determine the height and weight;
24. Patients who have large or medium-sized surgery, severe trauma, severe infection within 1 month before screening, and are not suitable for participation in this study as judged by the investigator;
25. Previous suicidality or suicidal behavior;
26. Anticipated surgery during the trial, except for outpatient surgery that is judged by the investigator to have no effect on subject safety and trial results;
27. Subjects with positive human immunodeficiency virus (HIV) antibody, hepatitis B surface antigen (HBsAg), hepatitis C (HCV) antibody or syphilis antibody at screening;
28. History of alcohol abuse within 1 month prior to screening. An average weekly alcohol intake of more than 21 units for men and 14 units for women, or unwillingness to stop drinking 24 hours before the day of medication and throughout the study (1 unit = 360 ml of beer, or 150 ml of red wine, or 45 ml of distilled spirits/liquor);
29. Positive urine screening test for drugs of abuse and drugs at screening;
30. Any laboratory test indicator meeting the following criteria (retests are allowed at screening with a clear reason, which should be recorded by the investigator):
31. Serum calcitonin ≥ 15 ng/L at screening;
32. Alanine aminotransferase ≥ 2.0 × ULN and/or aspartate aminotransferase ≥ 2.0 × ULN and/or total bilirubin ≥ 1.0 × ULN and/or alkaline phosphatase ≥ 2.0 × ULN at screening;
33. eGFR < 60 mL/min/1.73 m^2^ at screening, estimated using the CKD-EPI formula (see Appendix 2);
34. Abnormal thyroid function (FT3, FT4 or TSH) at screening;
35. Fasting triglycerides ≥ 5.64 mmol/L (500 mg/dl) at screening. If the patient is taking lipid-regulating therapies, the drug dose must be stable for 30 days before screening;
36. Serum amylase or lipase > 2.0 × ULN at screening;
37. The international normalized ratio (INR) of prothrombin time at screening is greater than the upper limit of the normal range;
38. Heart rate < 50 beats/min or > 90 beats/min on ECG at screening;
39. **The following clinically significant abnormalities in 12 electrocardiograms (ECGs) at screening: second-degree or third-degree atrioventricular block, long QT syndrome or QTcF > 450 ms (see Appendix 3 for calculation formula), PR interval < 120 ms or PR interval > 220 ms, QRS > 120 ms, left or right bundle branch block, Wolff-Parkinson-White syndrome or severe arrhythmia requiring treatment;**
40. Pregnant or lactating women and men or women of childbearing potential not willing to use contraception throughout the study;
41. Blood donation and/or blood loss ≥ 400 mL or bone marrow donation within 3 months before screening, or presence of hemoglobinopathy, hemolytic anemia, sickle cell anemia, or hemoglobin < 110 g/L (males) or < 100 g/L (females);
42. Subjects who, in the opinion of the investigator, have any other factors that may affect the efficacy or safety evaluation of this study and are not suitable for this study.

**Figure S1: Study design**


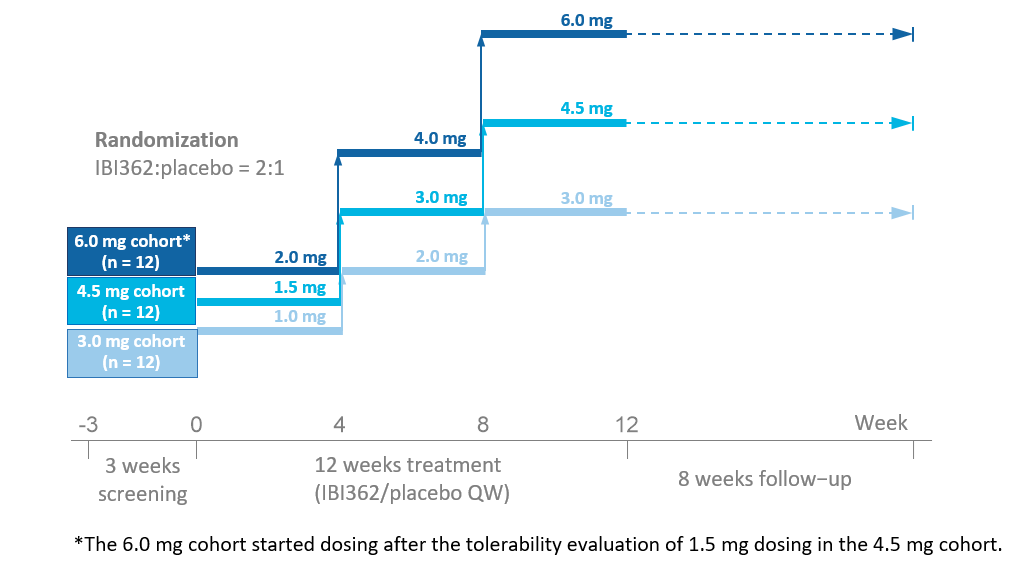


**Figure S2: By-week combined incidence (A) and onset time distribution (B) of gastrointestinal adverse events and decreased appetite**

Gastrointestinal adverse events included nausea, vomiting, diarrhea, abdominal pain, constipation, dyspepsia, abdominal distension, gastrointestinal inflammation and hiccups. GI = gastrointestinal.


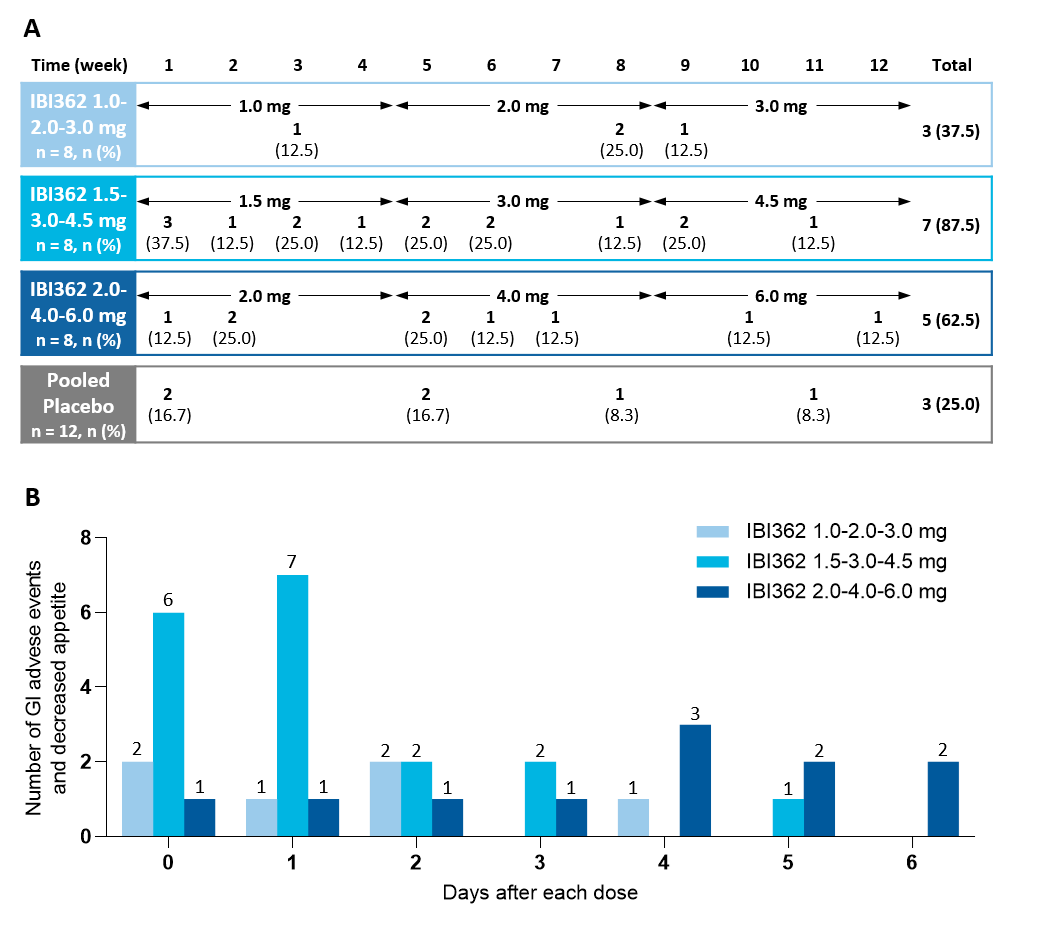


**Figure S3: Mean changes from baseline in heart rate (from standard 12-lead ECG), measured at 1 hour before each dose in the morning from week 0 to week 11, and 168 hours after the last dose (week 12)**

Error bars indicate SD. CFB = change from baseline. ECG = electrocardiogram.


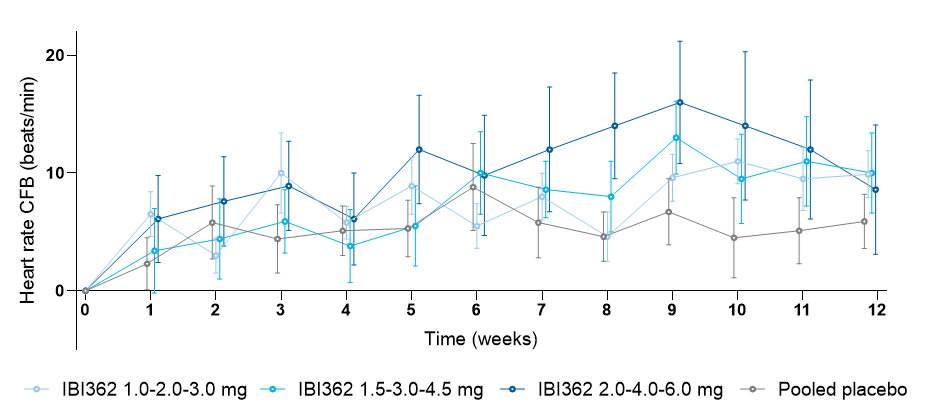


**Figure S4: Mean changes from baseline in lipase, amylase and calcitonin levels**

CFB = change from baseline. SE = standard error.


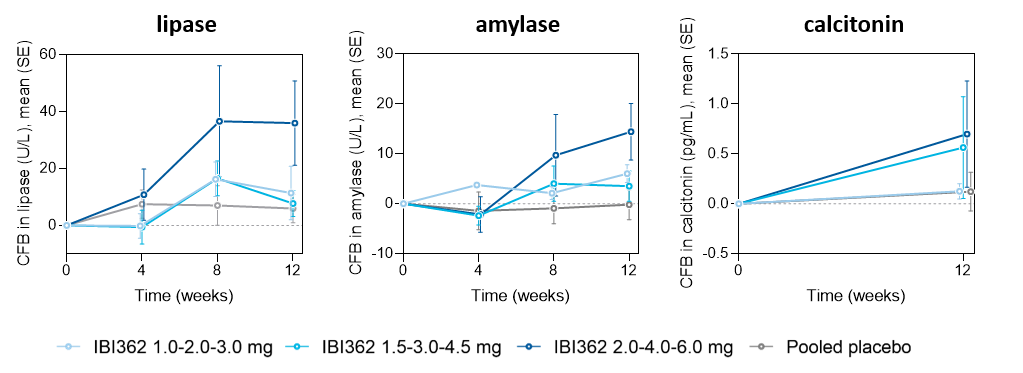


**Figure S5: Changes from baseline in serum uric acid and body weight** **for each participant**

CFB = change from baseline.


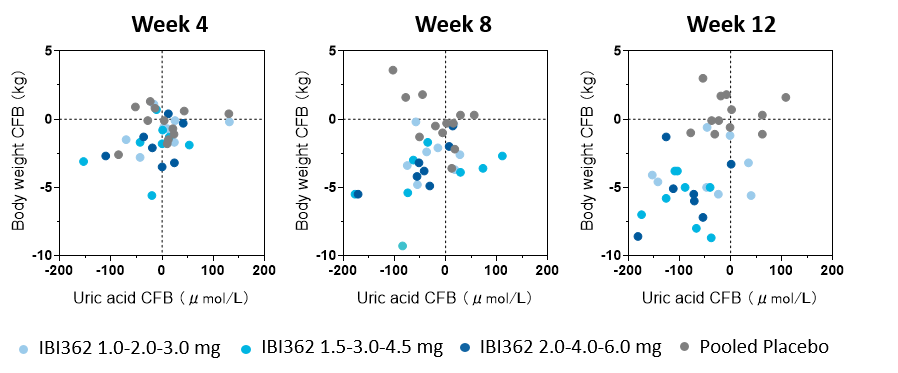


**Figure S6: Mean changes from baseline in ALT and AST levels**

CFB = change from baseline. ALT = alanine aminotransferase. AST = aspartate aminotransferase. SE = standard error.


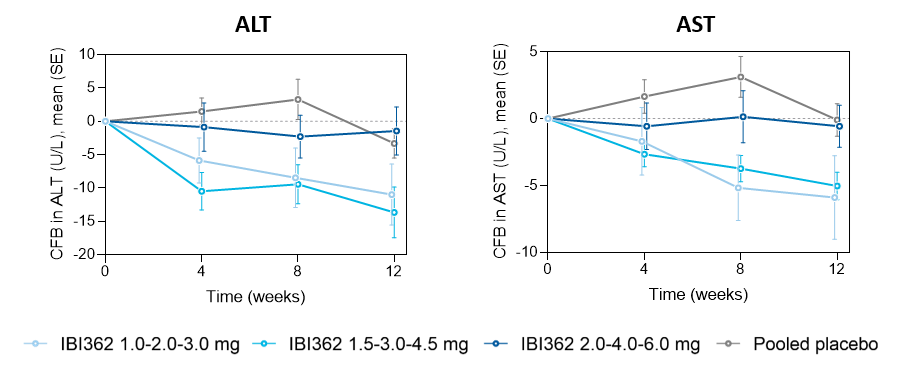


**Table S1: Distribution of participants among study centers**

|  | **3.0 mg cohort**  **(n = 12)** | | **4.5 mg cohort**  **(n = 12)** | | **6.0 mg cohort**  **(n = 12)** | | **Pooled placebo (n = 12)** |
| --- | --- | --- | --- | --- | --- | --- | --- |
|  | **IBI362**  **(n = 8)** | **placebo**  **(n = 4)** | **IBI362**  **(n = 8)** | **placebo**  **(n = 4)** | **IBI362**  **(n = 8)** | **placebo**  **(n = 4)** |  |
| Study centre |  |  |  |  |  |  |  |
| 02 | 0 | 0 | 2 | 0 | 2 | 1 | 1 |
| 04 | 2 | 1 | 0 | 0 | 2 | 0 | 1 |
| 05 | 2 | 1 | 1 | 0 | 2 | 0 | 1 |
| 07 | 3 | 1 | 3 | 0 | 1 | 1 | 2 |
| 08 | 0 | 1 | 1 | 2 | 0 | 2 | 5 |
| 09 | 1 | 0 | 1 | 2 | 1 | 0 | 2 |

Data are numbers of participants.

**Table S2: Treatment emergent adverse events**

| **System Organ Classification and Preferred Term** | **3.0 mg cohort**  **(n = 12)** | | **4.5 mg cohort**  **(n = 12)** | | **6.0 mg cohort**  **(n = 12)** | | **Pooled placebo (n = 12)** |
| --- | --- | --- | --- | --- | --- | --- | --- |
|  | **IBI362** | **placebo** | **IBI362** | **placebo** | **IBI362** | **placebo** |  |
|  | **(n = 8)** | **(n = 4)** | **(n = 8)** | **(n = 4)** | **(n = 8)** | **(n = 4)** |  |
| Individuals with ≥ 1 TEAE | 6 (75.0) | 2 (50.0) | 7 (87.5) | 2 (50.0) | 6 (75.0) | 3 (75.0) | 7 (58.3) |
| Gastrointestinal disorders | 4 (50.0) | 1 (25.0) | 5 (62.5) | 1 (25.0) | 4 (50.0) | 1 (25.0) | 3 (25.0) |
| Diarrhoea | 0 | 0 | 3 (37.5) | 1 (25.0) | 3 (37.5) | 0 | 1 (8.3) |
| Nausea | 0 | 0 | 3 (37.5) | 1 (25.0) | 1 (12.5) | 0 | 1 (8.3) |
| Abdominal pain | 0 | 1 (25.0) | 2 (25.0) | 0 | 0 | 0 | 1 (8.3) |
| Constipation | 1 (12.5) | 0 | 0 | 0 | 0 | 1 (25.0) | 1 (8.3) |
| Dry mouth | 0 | 0 | 2 (25.0) | 0 | 0 | 0 | 0 |
| Vomiting | 1 (12.5) | 0 | 0 | 0 | 1 (12.5) | 0 | 0 |
| Mouth ulceration | 1 (12.5) | 0 | 0 | 0 | 0 | 0 | 0 |
| Dyspepsia | 0 | 0 | 0 | 0 | 1 (12.5) | 0 | 0 |
| Gastrointestinal inflammation | 1 (12.5) | 0 | 0 | 0 | 0 | 0 | 0 |
| Abdominal distension | 1 (12.5) | 0 | 0 | 0 | 0 | 0 | 0 |
| Metabolism and nutrition disorders | 1 (12.5) | 0 | 6 (75.0) | 2 (50.0) | 3 (37.5) | 2 (50.0) | 4 (33.3) |
| Decreased appetite | 1 (12.5) | 0 | 5 (62.5) | 1 (25.0) | 1 (12.5) | 1 (25.0) | 2 (16.7) |
| Hyperuricaemia | 0 | 0 | 1 (12.5) | 0 | 1 (12.5) | 1 (25.0) | 1 (8.3) |
| Dyslipidaemia | 0 | 0 | 1 (12.5) | 0 | 0 | 0 | 0 |
| Starvation | 0 | 0 | 0 | 0 | 1 (12.5) | 0 | 0 |
| Hyperlipidaemia | 0 | 0 | 0 | 1 (25.0) | 0 | 0 | 1 (8.3) |
| Infections and infestations | 4 (50.0) | 0 | 2 (25.0) | 1 (25.0) | 1 (12.5) | 2 (50.0) | 3 (25.0) |
| Upper respiratory tract infection | 2 (25.0) | 0 | 2 (25.0) | 0 | 0 | 1 (25.0) | 1 (8.3) |
| Nasopharyngitis | 1 (12.5) | 0 | 0 | 1 (25.0) | 0 | 1 (25.0) | 2 (16.7) |
| Tonsillitis | 1 (12.5) | 0 | 0 | 0 | 0 | 0 | 0 |
| Folliculitis | 0 | 0 | 0 | 0 | 1 (12.5) | 0 | 0 |
| Investigations | 0 | 1 (25.0) | 1 (12.5) | 1 (25.0) | 0 | 1 (25.0) | 3 (25.0) |
| Blood glucose increased | 0 | 1 (25.0) | 0 | 1 (25.0) | 0 | 1 (25.0) | 3 (25.0) |
| Neutrophil count increased | 0 | 0 | 1 (12.5) | 0 | 0 | 0 | 0 |
| White blood cell count increased | 0 | 0 | 1 (12.5) | 0 | 0 | 0 | 0 |
| Cardiac disorders | 1 (12.5) | 0 | 2 (25.0) | 0 | 0 | 0 | 0 |
| Atrioventricular block first degree | 1 (12.5) | 0 | 0 | 0 | 0 | 0 | 0 |
| Myocardial ischaemia | 0 | 0 | 1 (12.5) | 0 | 0 | 0 | 0 |
| Sinus tachycardia | 0 | 0 | 1 (12.5) | 0 | 0 | 0 | 0 |
| Renal and urinary disorders | 1 (12.5) | 0 | 2 (25.0) | 0 | 0 | 0 | 0 |
| Proteinuria | 0 | 0 | 2 (25.0) | 0 | 0 | 0 | 0 |
| Calculus urinary | 1 (12.5) | 0 | 0 | 0 | 0 | 0 | 0 |
| General disorders and administration site conditions | 1 (12.5) | 0 | 1 (12.5) | 0 | 0 | 0 | 0 |
| Pyrexia | 1 (12.5) | 0 | 0 | 0 | 0 | 0 | 0 |
| Fatigue | 0 | 0 | 1 (12.5) | 0 | 0 | 0 | 0 |
| Injury, poisoning and procedural complications | 1 (12.5) | 0 | 1 (12.5) | 0 | 0 | 0 | 0 |
| Thermal burn | 0 | 0 | 1 (12.5) | 0 | 0 | 0 | 0 |
| Soft tissue injury | 1 (12.5) | 0 | 0 | 0 | 0 | 0 | 0 |
| Respiratory, thoracic and mediastinal disorders | 0 | 0 | 1 (12.5) | 0 | 1 (12.5) | 0 | 0 |
| Oropharyngeal pain | 0 | 0 | 1 (12.5) | 0 | 0 | 0 | 0 |
| Hiccups | 0 | 0 | 0 | 0 | 1 (12.5) | 0 | 0 |
| Reproductive system and breast disorders | 1 (12.5) | 0 | 0 | 0 | 0 | 1 (25.0) | 1 (8.3) |
| Menstruation irregular | 0 | 0 | 0 | 0 | 0 | 1 (25.0) | 1 (8.3) |
| Dysmenorrhoea | 1 (12.5) | 0 | 0 | 0 | 0 | 0 | 0 |
| Skin and subcutaneous tissue disorders | 1 (12.5) | 1 (25.0) | 0 | 0 | 0 | 0 | 1 (8.3) |
| Urticaria | 1 (12.5) | 1 (25.0) | 0 | 0 | 0 | 0 | 1 (8.3) |
| Musculoskeletal and connective tissue disorders | 1 (12.5) | 0 | 0 | 0 | 0 | 0 | 0 |
| Back pain | 1 (12.5) | 0 | 0 | 0 | 0 | 0 | 0 |
| Psychiatric disorders | 0 | 0 | 1 (12.5) | 0 | 0 | 0 | 0 |
| Mental fatigue | 0 | 0 | 1 (12.5) | 0 | 0 | 0 | 0 |

Data are presented as n (%)
